# A richly interactive exploratory data analysis and visualization tool using electronic medical records

**DOI:** 10.1186/s12911-015-0218-7

**Published:** 2015-11-12

**Authors:** Chih-Wei Huang, Richard Lu, Usman Iqbal, Shen-Hsien Lin, Phung Anh (Alex) Nguyen, Hsuan-Chia Yang, Chun-Fu Wang, Jianping Li, Kwan-Liu Ma, Yu-Chuan (Jack) Li, Wen-Shan Jian

**Affiliations:** 1 Graduate Institute of Biomedical Informatics, College of Medical Science and Technology, Taipei Medical University, Taipei, Taiwan; 2 International Center for Health Information Technology (ICHIT), Taipei Medical University, Taipei, Taiwan; 3 Institute of Biomedical Informatics, National Yang Ming University, Taipei, Taiwan; 4 Department of Computer Science, University of California-Davis, Davis, CA USA; 5 Department of Dermatology, Wan-Fang Hospital, Taipei, Taiwan; 6 School of Health Care Administration, Taipei Medical University, Taipei, Taiwan; 7 Faculty of Health Sciences, Macau University of Science and Technology, Macau, China

## Abstract

**Background:**

Electronic medical records (EMRs) contain vast amounts of data that is of great interest to physicians, clinical researchers, and medial policy makers. As the size, complexity, and accessibility of EMRs grow, the ability to extract meaningful information from them has become an increasingly important problem to solve.

**Methods:**

We develop a standardized data analysis process to support cohort study with a focus on a particular disease. We use an interactive divide-and-conquer approach to classify patients into relatively uniform within each group. It is a repetitive process enabling the user to divide the data into homogeneous subsets that can be visually examined, compared, and refined. The final visualization was driven by the transformed data, and user feedback direct to the corresponding operators which completed the repetitive process. The output results are shown in a Sankey diagram-style timeline, which is a particular kind of flow diagram for showing factors’ states and transitions over time.

**Results:**

This paper presented a visually rich, interactive web-based application, which could enable researchers to study any cohorts over time by using EMR data. The resulting visualizations help uncover hidden information in the data, compare differences between patient groups, determine critical factors that influence a particular disease, and help direct further analyses. We introduced and demonstrated this tool by using EMRs of 14,567 Chronic Kidney Disease (CKD) patients.

**Conclusions:**

We developed a visual mining system to support exploratory data analysis of multi-dimensional categorical EMR data. By using CKD as a model of disease, it was assembled by automated correlational analysis and human-curated visual evaluation. The visualization methods such as Sankey diagram can reveal useful knowledge about the particular disease cohort and the trajectories of the disease over time.

## Background

Electronic medical records (EMRs) are now widespread and collecting vast amounts of data about patients and metadata about how healthcare is delivered. These small datacenters have the potential to enable a range of health quality improvements that would not be possible with paper-based records [1]. However, the large amounts of data inside EMRs come with one large problem: how to condense the data so that is easily understandable to a human. The volume, variety and veracity of clinical data present a real challenge for non-technical users such as physicians and researchers who wish to view the data. Without a way to quickly summarize the data in a human-understandable way, the insights contained within EMRs will remain locked inside.

Many EMRs are also not flexible enough to accommodate the information needs of different types of users. For instance, clinicians often try to combine data from different information systems in order to piece together an accurate context for the medical problems of the patient who is in the room with them. Clinical researchers, however, may be primarily interested in finding population level outcomes or differences between cohorts. Administrators use EMR data to inform healthcare policy, while patients who use EMRs may be interested in comparing their health to their peers or tracking their own health over time [2]. Unfortunately, little support exists in current EMR systems for any of these common use cases, which hampers informed decision-making.

Visual analytics, also known as data visualization, holds the potential to address the information overload that is becoming more and more prevalent. Visual analytics is the science of analytical reasoning facilitated by advanced interactive visual interfaces [3, 4]. It can play a fundamental role in all IT-enabled healthcare transformation but particularly in healthcare delivery process improvement. Interactive visual approaches are valuable as they move beyond traditional static reports and indicators to mapping, exploration, discovery, and sense-making of complex data. Visual analytics techniques combine concepts from data mining, machine learning, human computing interaction, and human cognition. In healthcare, data visualization has already been used in the areas of patient education, symptom evolution, patient cohort analysis, EHR data and design, and patient care plans. This enables decision makers to obtain ideas for care process data, see patterns, spot trends, and identify outliers, all of which aid user comprehension, memory, and decision making [5].

Our objective is to create a visually interactive exploratory data analysis tool that can be used to graphically show disease-disease associations over time. That is, the tool presents how a cohort of patients with one chronic disease may go on to develop other diseases over time. The study used chronic kidney disease (CKD) as the prototype chronic disease, users could easily change the software tool to visualize a different disease. In the previous study, we have verified that such a system can significantly raise the efficiency and performance of practicing physicians and clinical researchers who desire to use EMRs for their research projects [6, 7]. Expected cohort trajectories are of great interest in clinical research. Our main task, then, will be to identify underlying chronic diseases and explore what happens over time to the being diagnosed patients and what comorbidities they develop over time.

## Methods

### System design

The system is designed based on data transformations that are required to perform longitudinal cohort studies. The transformed data are connected by a sequence of adjustable operators. The output results are shown in a Sankey diagram–style timeline, which is a particular kind of flow diagram for showing factors’ states and transitions over time. The visualization is driven by the transformed data, and the user feedback is directed to the corresponding operators, and completing the iterative process.

#### Data transformation

**Fig. 1:**
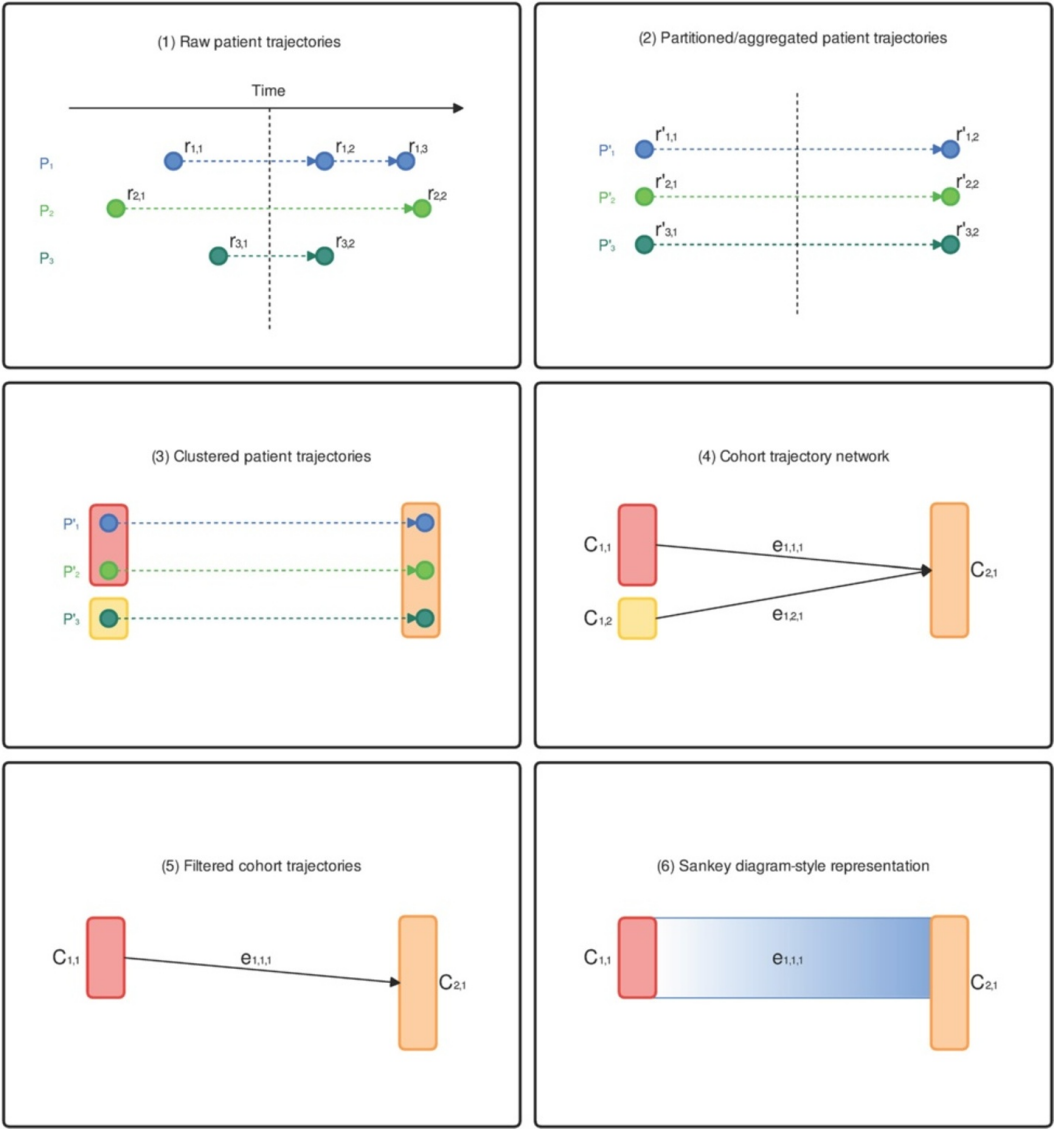
Data transformation processes. The data transformation steps behind the visual analysis process followed the analysis process from the raw patient records to the final visualization

The data transformation steps behind the visual analysis process are illustrated in Fig. 1. The transformation order follows the analysis process from raw patient records to the final visualization. Assume that there are *N* patients and *M* unique factors. As the top-most chart shows, the raw sequence of a patient can be treated as a discrete trajectory with non-uniformly distributed records along the time axis. We define the patient trajectories as P = {*p*_1_, …, *p*_*n*_, …, *p*_*N*_} and the set of factors as F = {*f*_1_, …, *f*_*m*_, …, *f*_*M*_}. A patient trajectory is an ordered sequence of *K*_*n*_ records: ${p}_n=\left({r}_{n,1},\dots, {r}_{n,k},\dots, {r}_{n,{k}_n}\right)$, where each record consists of a factor set and a timestamp: *r*_*n*,*k*_ = (*F*_*n*,*k*_, *t*_*n*,*k*_), *F*_*n*,*k*_ ⊂ *F*. Note the timestamp of each record is relative and not necessarily the actual record date. In the cohort study, we are interested in the temporal and populational patterns on the course of CKD. Therefore, it makes more sense to align each patient trajectory by their days before and after being diagnosed with CKD.

When the user specifies the time windows: T = (*t*_1_, …, *t*_*l*_, …, *t*_*L*_), the patient trajectories are partitioned based on their timestamps. Records in the same time window are merged into one:

$$ r{\hbox{'}}_{n,l}=\left(F{\hbox{'}}_{n,l},{t}_l\right) $$

$$ F{\hbox{'}}_{n,l}={\displaystyle \underset{i\in I}{\cup }{F}_{n,i}} $$

$$ \mathrm{I}=\left\{k\Big|{t}_l\le {t}_{n,k}<{t}_{l+1}\right\} $$

The end results are patient trajectories regulated in time, $P{\hbox{'}}_n=\left(r{\hbox{'}}_{n,1},\dots, {r^{\hbox{'}}}_{n,l},\dots, r{\hbox{'}}_{n,{L}_n}\right)$, where the timestamps are regulated by the time windows, and each record’s factor set represents all the factors observed on that patient within the time window. When the user requests for patient clustering, the patients at each time window are clustered based on a certain similarity measure and become a set of cohorts: ${C}_l = \left\{{c}_{l,1,},\dots, {c}_{l,h},\dots, {c}_{l,{H}_l}\right\}$, where *C*_*l*_ ⊂ Ρ and it represents a set of *H*_*i*_ cohorts at time window *t*_*l*_.

We define the cohort trajectory network as *G* = (*V*, *E*), where each node *V* = (*v*_*l*,*h*_|*v*_*l*,*h*_ = *c*_*l*,*h*_) represents a cohort at a time window, and each edge Ε = {*e*_*l*,*i*,*j*_|*v*_*l*,*i*_ → *v*_*l* + 1,*j*_, |*c*_*l*,*i*_ ∩ *c*_*l*,*j*_| > 0} represents the association between two cohorts at consecutive time windows where their members overlap. The network *G* is used to drive the visualization in the end of the process.

#### Data & control flow

**Fig. 2:**
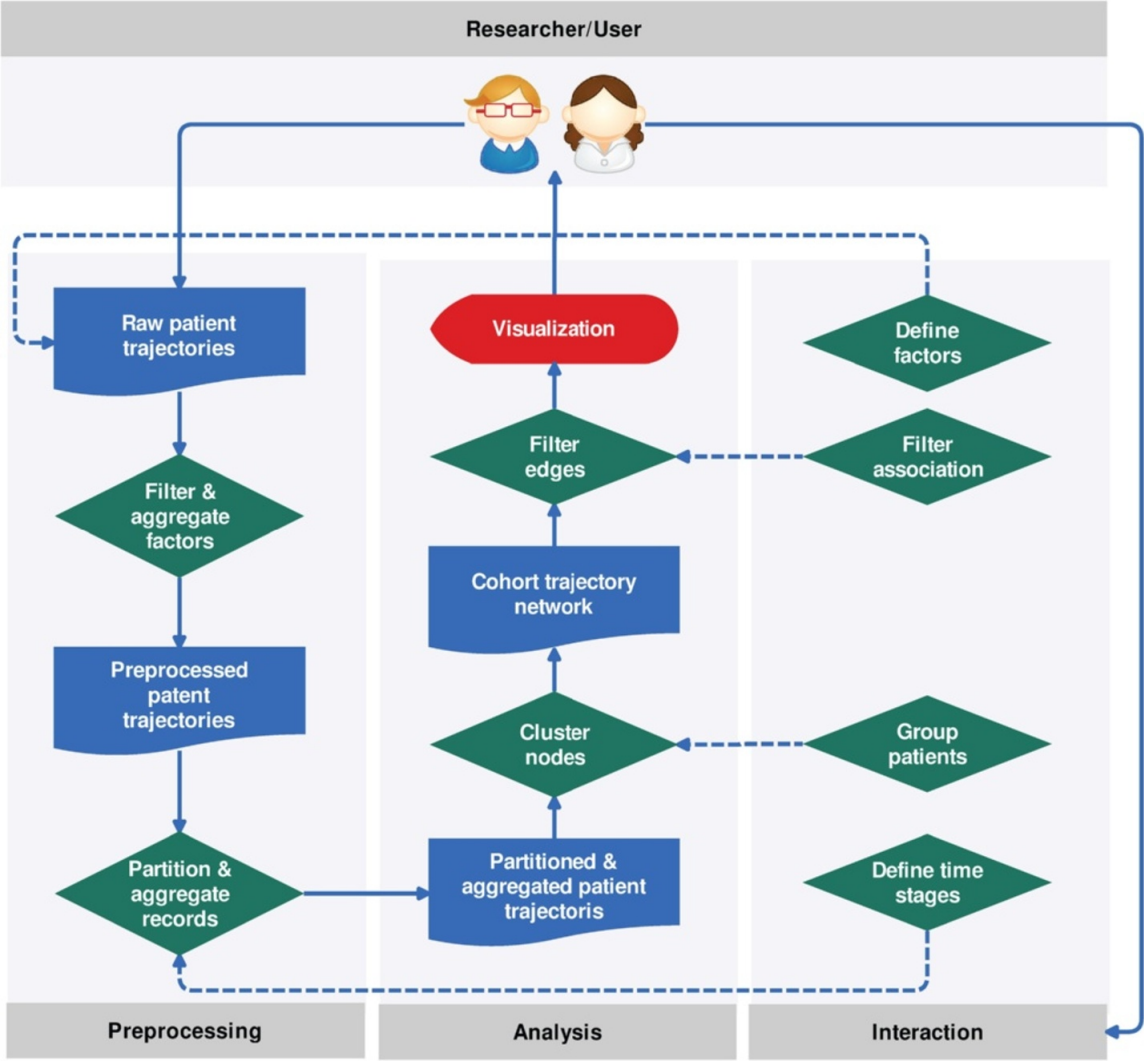
The data and control flow of visual analysis process from user perspective. The data flows through a sequence of operators, which were adjustable and associated with different interactions by the user

As shown in Fig. 2, data flows through a sequence of operators, which are adjustable and associated with different interactions by the user. The interaction workflow is designed from the user’s point of view, and it implements.

Once the user specifies the important factors for the study, the system scans the raw patient trajectories record by record, filters, and aggregates the factors accordingly. Similarly, the time windows defined by the user also changes the way the system partitions and aggregates the trajectories over time. The two operators, cluster nodes and filter edges, implement multiple techniques to support the analysis tasks of finding cohort and filtering associations, respectively. It is important to note that there is no once-and-for-all operation for any analysis task. Each cluster or filter operator has its strengths and its limitations, these are the reasons why we should be carefully employed.

(1) Frequency-based Cohort Clustering: Frequency-based clustering allows one to follow one’s basic intuition to see the “main idea” of data. Cohorts with higher cardinalities are preserved while minor ones are considered less important and merged. Our system allows the user to specify a threshold x for the cardinality, and it merges cohorts of sizes less than the threshold into the “others” group.

$$ cluster\left({C}_l\right)=\left\{\begin{array}{c}{c}_{l,h}\kern.7em  if\left|{c}_{l,h}\right|\ge x\hfill \\ {}\hfill others\  if\ \left|{c}_{l,h}\right|<x\ \hfill \end{array}\right. $$

(2) Hierarchical Cohort Clustering: Given a time window, each patient is characterized by the comorbidity of factors within the window. We consider the similarity between two unique comorbidities as the set relation of their factors. For example, two sets of factors {*f*_1_} and {*f*_1_, *f*_2_} are partially overlapped by the common factor *f*_1_. In consideration of such similarity, we apply hierarchical clustering to extract cohorts with similar comorbidities.

The resulting clusters are hierarchical and the user can specify the desired number of clusters. With more clusters one is able to describe the characteristics of each cohort more accurately, but more clusters introduce more nodes, more associations, and thus higher visual complexity. On the other hand, fewer clusters create less visual complexity at the expense of potentially overlooking some essential but smaller structures.

Given the set of factors: *s*_*i*_ = *F* ' _*i*,*l*_ at a time window *t*_*l*_ for a patient *p*_*i*_, we define the similarity between two patients with the Ochiai coefficient [8], which is a variation of cosine similarity between sets:

$$ similarity=\frac{\left|{s}_1\cap {s}_2\right|}{\sqrt{\left|{s}_1\right|\left|{s}_2\right|}} $$

(3) Variance-based Association Filtering: The importance of an association lies in how confident we are able to make an inference from it. We can extract the statistically important associations by ranking and filtering their variances. Our system demonstrates this capability by adopting one particular type of variance, which is defined as the outcome entropy of the associated cohort. Such entropy can be calculated by the conditional probabilities of the different outcomes of the given cohort:

$$ pb\left(p\in {c}_{l+1,j}\Big|p\in {c}_{l,i}\right)=pb\left({c}_{l+1,j}\Big|{c}_{l,i}\right)=\frac{\left|{c}_{l,i}\cap {c}_{l+1,j}\right|}{\left|{c}_{l,i}\right|} $$

$$ entropy\left({e}_{l,i,j}\right)=-{\displaystyle \sum_k}\left(pb\left({c}_{l+1,k}\Big|{c}_{l,i}\right)*pb\left({c}_{l+1,k}\Big|{c}_{l,i}\right)\right) $$

We can see that the entropy is minimized when the patients in a cohort at the current time window all go to another cohort at the next window. In contrast, it is maximized when the probabilities of patients who go to other cohorts are uniformly distributed. Our system allows filtering important associations by adjusting the entropy threshold. When the threshold is high, all associations are shown in spite of their variance; in the extreme case when the threshold is zero, only the associations of zero entropy will be displayed; in other words, it only visualizes the associations between fully overlapped cohorts.

#### Visualization design

Our system visualizes the cohort trajectories network model that we discussed in the previous section and summarizes it. The user can use it to assess important features such as cohort comorbidity, cohort distributions, and their associations across time windows, etc. We design the visual encoding and the optimization strategies in a way to maximize the legibility of the presentation.

(1) Visual Encoding: We encode the dimensions of the visual space similarly to OutFlow, where the x-axis encodes the time information and the y-axis is used for laying out the categories (comorbidities) [9]. We also visualize the associations between the cohorts as ribbons.

The visualization must convey the characteristics of both the cohorts and the associations. It is common to encode cardinality to the nodes and edges [10, 11] as such information allows the user to assess the frequency-based distribution. Our system encodes cardinality as the nodes’ or edges’ height. Each cohort is labeled to show its dominant characteristics. It lists the common factors shared by all patients in this group. If there are factors not shared by the entire group, we indicate it by appending an asterisk to the label. In addition, we map colors to unique comorbidities and assign each node its corresponding color. The edge color is determined by the two nodes it connects, and we use gradients for smooth transitions.

The visual encoding of our system is tailored for the CKD cohort study; however, it can be easily changed to display other relevant information. For example, instead of showing the cardinality, the edge can encode other statistical measurements that reveal set relations [12].

(2) Optimization: The overlap between cohorts could be complex and thus increase the number of edges as well as the number of edge crossings. It could impact the legibility of the visualization. Since the y-axis is nominal and the ordering between the categories is flexible, we can arrange the node’s vertical positions to reduce the amount of crisscrossing and thus resolve visual clutter.

The algorithm we apply to minimize edge crossing is modified from an existing library and is a heuristic iterative relaxation method [13]. The algorithm sweeps back and forth along the x-axis and adjusts the node vertical positions based on two objectives: (1) minimize the edge length, and (2) resolve node overlaps. It utilizes simulated annealing, so the process ends in a predictable time. The result is an approximation but the algorithm allows us to get reasonable results in an interactive rate.

In addition, the z-ordering (front to back of the screen) of the edges should be considered as well in order to maximize legibility [11]. We choose to place smaller edges on top of the larger ones to reveal the outliers.

#### Interaction methods

The system interface consists of two views: trajectory view and summary view. The trajectory view is time-based and displays an overview of patient trajectories that the user can interact directly with. It also highlights the trajectories of selected patients. Summary view presents the characteristics of the selected patient group. For example, it shows the distributions of gender, age, and factors, etc. It is also interactive and provides additional functions such as querying by patient metadata information.

Most data items (patients, factors, etc.) in the system are selectable, and the system automatically searches for related items and highlights such associations with visual links. For example, the user can select a cluster of patients by clicking on a node or an edge in the trajectory view. The patients selected are highlighted as red regions in each node and link. The highlighted regions also encode the cardinality as heights so it shows the proportion of the patients selected comparing to others. In the meantime, the highlighted edges reveal the paths traveled by the selected patients. In addition, the user can also select a factor, and all patients having this factor will be highlighted. This enables the user to observe the global distribution of a particular factor.

### Pilot study

#### Data sources

The original data source for this paper is from Taiwan’s National Health Insurance Research Database (NHIRD), a longitudinal database which contains International Classification of Disease, Ninth Revision, Clinical Modification (ICD-9-CM) codes for disease identification as well as procedure codes. The database contains health information for one million people over 13 years (1998–2011). We extracted 14,567 CKD patients who had eleven common comorbidities.

**Fig. 3:**
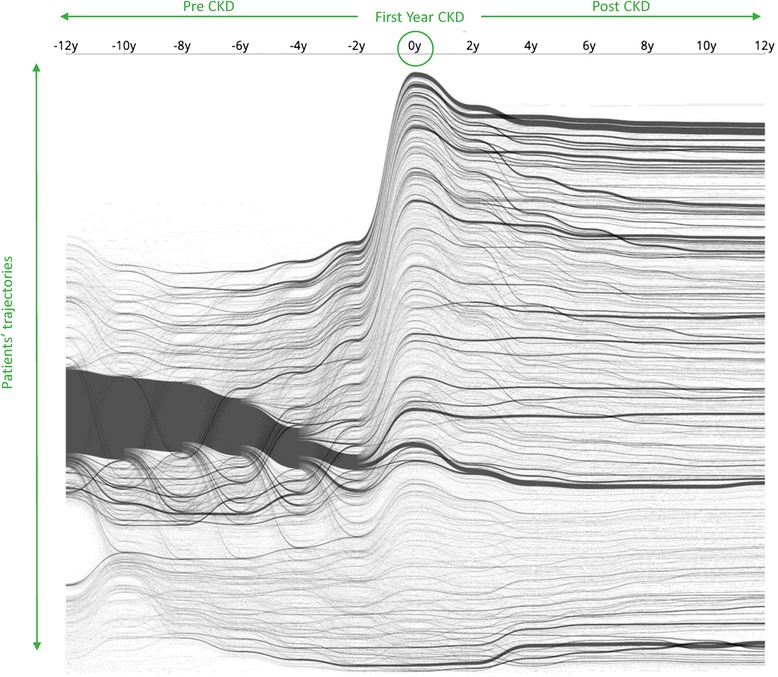
Time course of 14,567 CKD patients clustered by comorbidities. 14,567 CKD patients clustered according to comorbidities on the timeline. The x-axis showed the timeline covering 12 years before and after each patient who got a diagnosis of CKD, while the y-axis presents the clusters of trajectories for each CKD patient

Preparing to visualize clinical data involves a series of logical steps [4, 14]. The first step in the data visualization process is selecting the patient cohorts. Figure 3 shows the visualization with only 17 observed factors. The x-axis shows the patients’ conditions over the timeline before and after getting CKD diagnosis; the y-axis presents the arrangement of trajectories for each CKD patient, which they were aggregated together with the same comorbidity clusters. However, the outcome of visualization was too difficult to interpret and understand. The tool would be more useful for users if we could provide selection and aggregation function to associate their target patient groups.

**Fig. 4:**
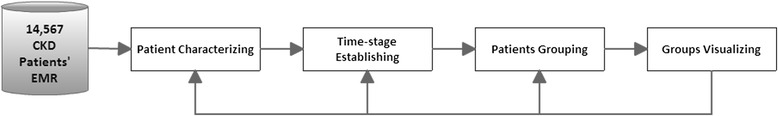
Classifying patients into uniform cohorts. The flow showed an overview of the data analysis process in this study. The visual analysis process was based on CKD research dataset

Another challenge in the cohort identification process is to standardize the large diversity and inhomogeneity of comorbidities in the database [4]. Due to those high-dimensional data, such as electronic medical records, would lower the homogeneity between data items, we used a divide-and-conquer approach to classify patients into relatively uniform within each groups. Figure 4 shows an overview of this process.

#### Factors

A factor is a general term used to describe a single criterion that is used to separate patients into cohorts. The factors are derived from diseases and procedures and are the fundamental elements that characterize a patient in our system. In the CKD cohort study, there are tens of thousands diseases and procedure codes that one could use to separate CKD patients. Defining the right set of factors is not a trivial task because including unnecessary factors that are either redundant or irrelevant to the analysis objectives increases the computational cost as well as jeopardizes the interpretability of the visualization. Our system is flexible enough to allow the user to define a set of factors by selecting independent ICD-9 codes or aggregating correlated ones based on the user’s domain knowledge. In this study, we worked with nephrologists to define 17 related criteria that users can visually explore concerning chronic kidney disease (Table 1). The 17 factors represent the most related diseases and procedures that follow a diagnosis CKD.

**Table 1:** Factor association rules

Diseases / Procedures (Abbreviation)	ICD-9 / Procedure code
Cerebrovascular Disease(CVD)	430–438
Chronic Kidney Disease(CKD)	585, 586
Congestive Heart Failure(CHF)	398.91, 402, 404, 425.4-425.9,428
Coronary Artery Disease(CAD)	410–414
Diabetes mellitus(DM)	250
Glomerulonephritis(GN)	582
Hemodialysis(HD)	58001C,58019C,58020C,58021C,58022C,58023C,58024C,58025C,58027C,58029C,58030B
Hyperlipidemia	272
Hypertension(HTN)	401
Peritoneal Dialysis(PD)	58002C,58009B,58010B,58011C,58012B,58017C,58028C
Polycystic Kidney Disease(PKD)	75312
Proteinuria	791
Renal stone	592
Renal transplantation(RTPL)	V420
Systemic Lupus Erythematosus(SLE)	7100

#### Time windows

Visualizing EMR data over time also requires the ability to change the granularity of the x-axis (time). For example, in CKD there are several stages in its natural history. Within each stage, CKD can be relatively stable, but there is inhomogeneity between CKD patients at different stages. Therefore, we use time windows to refer the time duration or interval (i.e., 1-month, 1-year, 2-year…etc.) To decide on a time granularity is a manual process that is often best judged by humans [3]. The results are patient trajectories partitioned over time, which accentuates the differences between cohorts.

#### Patient groups

While patient comorbidities within each time window are expected to be stable, comorbidities are not stable over the entire population across all time windows.

Our system handles this problem by using clustering methods which make clear the underlying comorbidity distributions within each patient group. The end results are cohorts that have reliable distributions of comorbidities.

#### Visual examination

Once the time windows are defined and the cohorts are extracted, the quality of the visualization can be evaluated by examining the associations between cohorts. For instance, the user might want to examine how cohorts merge or diverge over time. Our system reveals not just associations that would otherwise be impossible a person to notice, but also allows users to interact with the underlying data immediately to facilitate “what-if” scenarios. Sometimes, however, the quantity or variance of the associations could be large and thus lead to visual clutter problems. Therefore, our system also allows the user to rank and filter the associations based on their statistical importance. This way, the user can limit themselves to exploring visual changes that are also in fact statistically significant both visually and mathematically. At any step of the visual analysis process, the user can go back and change the settings for factors, time windows, patient clustering, and comorbidity association filtering. For example, if the user wants to explore the temporal patterns in finer detail and examine if there are local and short-term patterns, the user can add more time windows to the context; on the other hand, if two or more stages exhibit indistinguishable patterns, the user might want to merge those time windows as they do not convey extra information. The user can also change the parameters to refine how patients are grouped or how associations are filtered. This iterative process continues until the user obtain a result that he/she is satisfied with.

We use the CKD as a model chronic disease to demonstrate the analysis process, but the process can be applied to the study of other diseases as well. For example, if the user wants to study the clinical trajectories of diabetics, the user can define a list of factors related to diabetes. Then the user can apply the same process to set up time windows, cluster patients, and explore cohort trajectories.

### Ethical approval

This type of study was not required the Institutional Review Board review in accordance with the policy of National Health Research Institutes which provides the large computerized de-identified data (http://nhird.nhri.org.tw/en/).

## Results

### Exploring cohort structures

In this study, we build an exploratory data analysis tool that depicts the trajectories of 14,567 CKD patients’ comorbidities over time. We partition the records into multiple 2-year time windows. Researchers often have different factors-of-interest for different windows of CKD. In the pre-CKD stage, they are interested in common diseases such as hypertension, diabetes; for end-stage CKD factors, they are interested in critical procedures such as dialysis, renal transplantation, or patient death. We filter the factors of interest according to each time window.

**Fig. 5:**
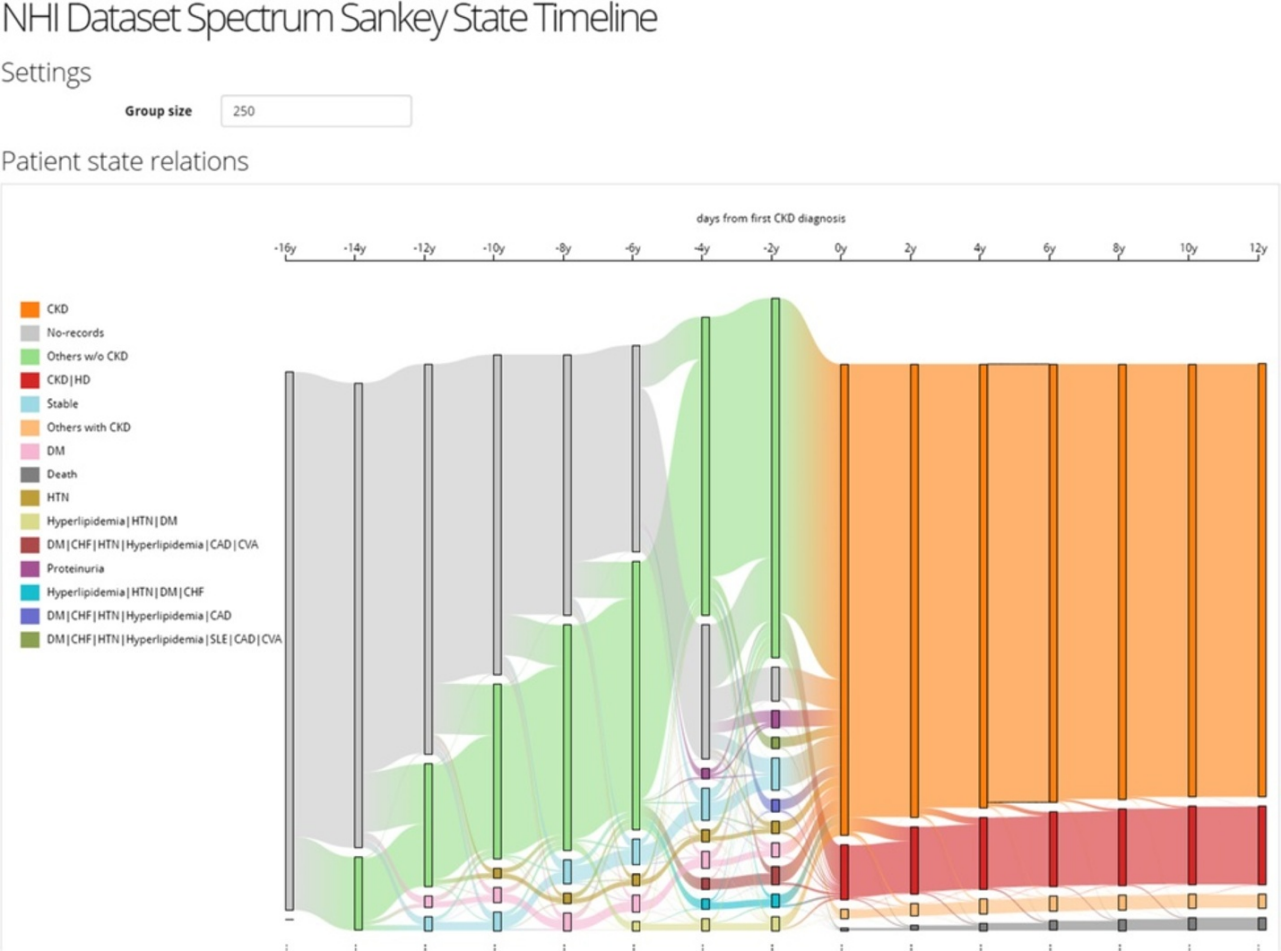
Frequency-based Cohort Clustering: Sankey Diagrams for CKD Cohort Sizes of < 250. The trajectories were simplified where larger cohorts were kept and smaller ones were merged into a single “others” group. The light green for others without CKD and light orange for others with CKD

**Fig. 6:**
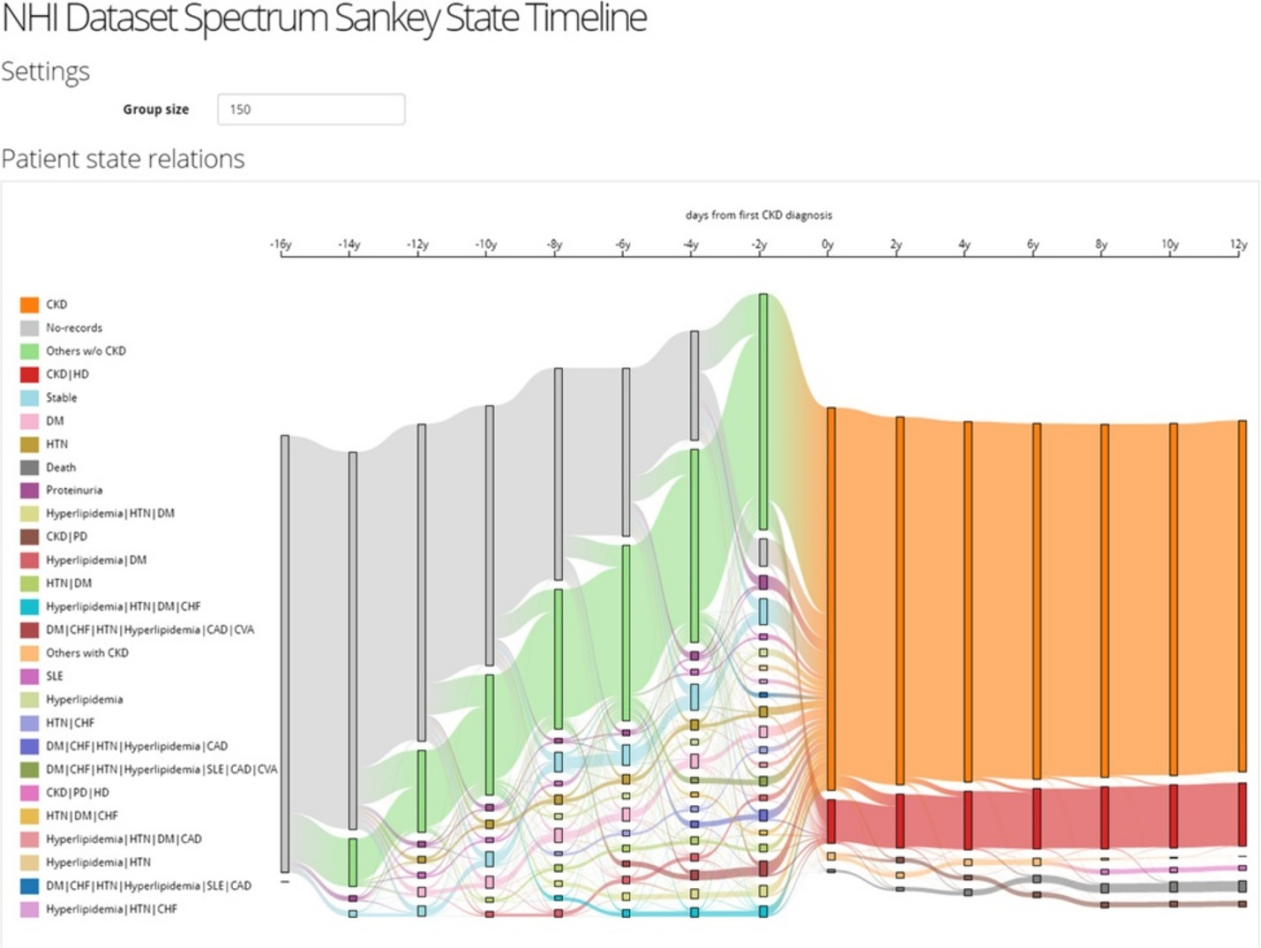
The system visualization displayed with the threshold adjust to 150. The user could enlarge/lower the threshold to reveal different size of cohorts

Since there are too many comorbidities to visualize clearly as shown in Fig. 3, we apply frequency-based cohort clustering to extract the dominant cohorts. As Fig. 5 shows, the trajectories are simplified where larger cohorts are kept and smaller ones are merged into a single “others” group (light green for others without CKD and light orange for others with CKD). From the overviews, we can learn about the prevalence of different comorbidities and their proportions in the population. For example, we can see from Fig. 5 that the number of patients with a single disease such as hypertension (HTN) (brown) and diabetes (DM) (dark blue) shrinks as the time approaches year 0, which means that patients start to exhibit other diseases. The user can lower the threshold to reveal smaller sized cohorts as shown in Fig. 6.

### Exploring associated relationships

Another goal of exploratory data analysis is to uncover unexpected associations between two variables. In this study, we demonstrate the exploring associations between hemodialysis (HD) in early stages of CKD and other diseases and procedures. More specifically, we want to identify the driving factors that may lead to hemodialysis and the downstream consequences.

First, we divide CKD patients according to CKD severity: (1) pre-CKD: before the patient’s first CKD diagnosis, (2) first year-of-CKD, and (3) post-CKD: a year after the patient’s first CKD diagnosis. Second, we filter CKD patients according to pre-determined criteria that nephrologists determined to be clinically important. For the first-year-of-CKD stage, we focus on which patients will go on to require hemodialysis; for the post-CKD stage, we watch other common diseases and procedures related to CKD patients: Death, peritoneal dialysis (PD), and renal transplant (RTPL); for the pre-CKD stage, we watch all 17 diseases/procedures. As a result, there are 835 unique combinations at the pre-CKD stage, two at the first-year-of- CKD stage and nine at the post-CKD stage.

**Fig. 7:**
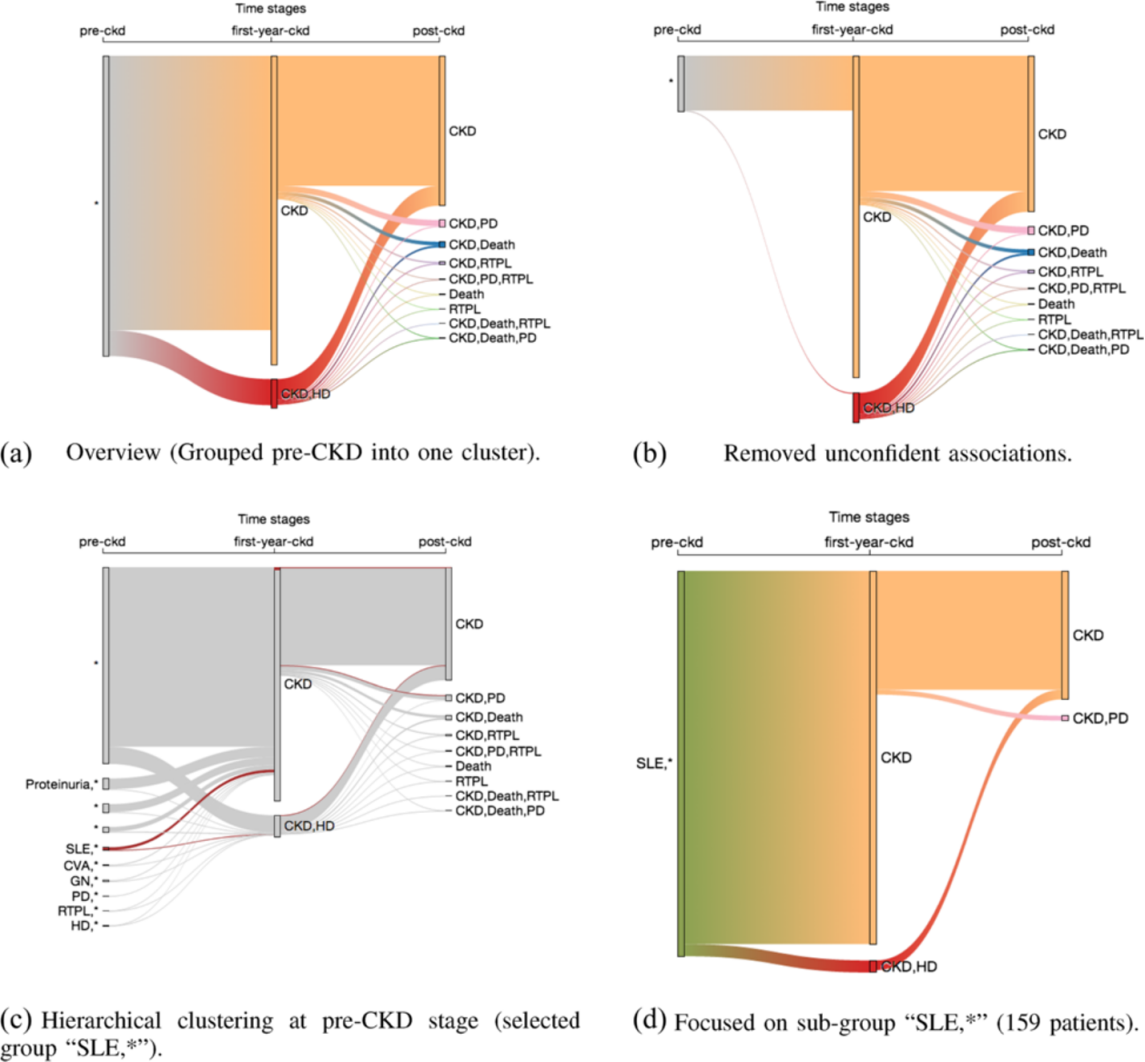
Explore causal relationship (12,960 patients). **a** There were 70.2 % of the patients who took HD in the first year of CKD did not develop any other factors, while the rest of them either took PD, RTPL, or died. **b** After filtering the unconfident associations, the remaining associations only covers 17.4 % of the population. **c** To perform hierarchical clustering on the patients at the pre-CKD stage and generate ten groups of similar patients. **d** When we highlighted the group who had a common factor of systemic lupus erythematosus (SLE), we found that none of them took the more serious procedures such as renal transplantation or died. Note: There are three groups labelled “*” because of the groups have no common factor shared by all members in the group

Since there are only a total of 11 CKD/disease or CKD/procedure combinations for first-year-of-CKD stage and the post-CKD patients, we can visualize their clinical courses without any simplification processes. However, there are too many combinations at the pre-CKD stage to be visualized directly. For simplicity, we first group them into one single cluster and focus on the last two time windows. As Fig. 7a shows, we find that 70.2 % of the patients who took hemodialysis in the first year of CKD did not develop any other diseases or procedures related to CKD, while the rest of them either required peritoneal (PD) or renal transplantation (RTPL), or died. Some of the patients who were not on hemodialysis in the first year also died; however, the mortality rate seems lower. We also notice that more than half of the patients who didn’t require hemodialysis in the first year are not associated with any of post-CKD factors of interest. This means they were either in stable condition after the first year or their following treatments were not recorded.

To see stronger associations between the pre-CKD factors and HD during the first-year-of-CKD stage, we must filter out the associations that are not helpful. For example, if a group of similar patients are associated with both “CKD” and “CKD|HD” clusters, it’s hard to tell whether this combination of factors will lead to hemodialysis or not. We can rule out all those unconfident associations by filtering according to the variance of their associations. We set a strict threshold 0.0 for the variance so that the association is kept only when it is 100 % confident. After the filtering, 32.6 % of the 835 unique combinations are taken out because their associations with the first-year-of-CKD stage are not confident. Figure 7b shows that the remaining associations only covers 17.4 % of the population. This means the pre-defined 17 factors might not be good explanatory variables to discriminate patients taking or not taking hemodialysis in the first year of CKD.

Next, we perform hierarchical clustering on the patients at the pre-CKD stage and generate ten groups of similar patients, as shown in Fig. 7c. Note there are three groups labeled “*”, which seems confusing at first as they could have been merged into one group. In fact, the three groups have different factor distributions. They are labeled “*” because none of the groups have a common factor shared by all members in the group. To avoid confusion, the user can assign a custom label to describe the nature of the group. When we select and highlight the group who has a common factor of systemic lupus erythematosus (SLE), we find that none of them required more serious procedures such as renal transplantation or died. Figure 7d is a zoom-in view showing the structure of the selected “SLE,*” group. We also notice that the proportion of patients requiring hemodialysis in the first year of CKD in the “SLE,*” group (3.14 %) is one-third of the proportion in the entire population (9.54 %).

### Responsiveness

Our system is a web-based (http://sankey.ic-hit.net/) and is tested with a commodity desktop machine (CPU: 2.66 GHz Quad-Core, Memory: 8GB 1066 MHz DDR3) as the application server and another desktop machine as the client. Most of the back end programs are written in Python, and the front end programs are written in Javascript and HTML5.

The system caches the transformed data after each operation in the data control flow (as shown in Fig. 2) to reduce unnecessary processing time and improve user end responsiveness. There are four major types of user interactions: defining factors, partitioning time windows, merging patients and filtering associations. The first two interactions usually happen at the beginning of a study and occasionally happen in major revisions. On the other hand, the rest of two interaction types are much more frequent in the analysis process. Caching the less frequently updating results helps us reduce unnecessary processing time.

We measure the time elapsed for each process using the system timer. For 14,567 patients and 6,031,579 records, it takes 6 min to filter and aggregate factors of the entire data set, and 25 s to partition the data set into three time windows. However, such operations are taken only a few times throughout the analysis and thus do not require immediate response. More frequently performed operations such as clustering patients or filtering associations only take 5 s per time window on average.

## Discussion

We present a system to visually analyze the comorbidities associated with CKD by using a large-scale database containing 14,567 patients. We visualize the results using a Sankey diagram to help practicing physicians and clinical researchers investigate the outcome of this complex disease based on comorbidities or procedures that these patients have.

Building a visually interactive exploratory data analysis tool is not without several challenges. First, direct visualization of all the patients can easily lead to overplotting. Second, in this dataset, there exists tens of thousands of risk factors pertinent to CKD patients. It is not apparent how to best discriminate and visualize these factors to bring out structures of interest in the data. After all, one of the main goals of data visualization is to bring out unexpected patterns in the data, which is best achieved by unsupervised machine learning methods. Figure 3 shows an unfiltered visualization of the CKD and 17 associated comorbidities and procedures. You may find out that the visualization is too complex to comprehend. It would be useful to select, aggregate, and visualize factors associated with patient groups. We have developed an interactive visualization system to support such operations.

### Temporal visualization

Time-series information are traditionally of particular interest when analyzing EMRs. [15] Much prior work has suggested presenting patient history longitudinally [16–18]. Real world data usually has prohibitively high visual complexity due to its high dimensionality or high variance. Thus, several simplification methods have been proposed. Bui et al. suggested using folder as well as non-linear spacing [19]. In the V-model project, Park et al. compressed the causality relationship along a linear timescale to an ordinal representation to carry more contextual information of the event [20]. In addition to abstracting time to use the horizontal screen real estate more efficiently, there are methods to save the vertical real estate. Bade et al. implemented a level-of-detail technique that presents data in five different forms based on its source and the row height available [21]. Our method simplifies the visual complexity of patient trajectories by aggregating records over time, clustering patients and filtering associations between cohorts.

### Query-based visual analytics

In many real world cases, the user can narrow down the scope and reduce the complexity of the data by querying based on his or her domain knowledge. Systems of this kind allow the user to specify the pattern of interest and can enhance the analytic process with advanced interfaces [22, 23]. However, it is not always easy to translate an analysis task into proper queries [24]. For temporal event queries, Wang et al. proposed an interactive system to support querying with higher level semantics such as precursor, co-occurring, and aftereffect events [25]. Their system outputs visual-oriented summary information to show the prevalence of the events as well as to allow comparison between multiple groups of events [26]. For overview specific tasks, Wongsuphasawat et al. proposed LifeFlow, a novel visualization that simplifies and aggregates temporal event sequences into a tree-based visual summary [27]. Monroe et al. improved the usability of the system by integrating interval-based events and developing a set of user-driven simplification techniques in conjunction with a metric for measuring visual complexity [13, 28]. Wongsuphasawat et al. also extended LifeFlow into a Sankey diagram-based visualization, which reveals the alternative paths of events and helps the user understand the evolution of patient symptoms and other related factors [29]..

In spite of their effectiveness in guided or well-informed analysis, query-based systems fall short for exploratory analysis where the user may not have a well-defined hypothesis and simply wants to explore and learn the data.

### Exploring inhomogeneous data

High-dimensional data items are less homogeneous and harder to compare with each other. It is harder to associate, rank, or filter those items meaningfully. Some have proposed that data be sliced and diced by dimension or item and separated into homogeneous subsets [4]. It has been proven that, by carefully selecting projection methods, a system can incorporate multiple heterogeneous genetic data and identify meaningful clusters of patients [30]. Our work is an example of the slice-and-dice concept, where we partition the record time into multiple dimensions and group patients within each time window.

We would like to investigate the possibility of using more sophisticated feature extraction methods in future work. In this case, we define the factors by hand with domain knowledge and group the patients based on the factors by a simple set similarity metric or a frequency-based metric. However, the combinations of factors are noisy and the variance within each cluster are usually high. Furthermore, there are still thousands of unused factors that may provide additional insights. Such problem could potentially be addressed with the help of correspondence analysis.

More optimizations can also be made to enhance the visual rendering of information as well. First, for conveying the association between the clusters, in this work we only visualize the cardinality of the association and filter them by variance. There are other measures of proportionality available which can help evaluate the association of comorbidities [31]. We would like to study each method’s role and effectiveness by conducting different analysis tasks. Second, for conveying and comparing the nature of each cluster, in this work we only present such information as text that shows the dominant factors of the cluster and indicate uncertainty. However, the underlying differences are non-binary and high-dimensional. Getting the system to effectively extract and present the subtle differences between the clusters could be the key to improving visual pattern depiction.

Finally, it is possible to improve the computational performance by parallel data processing. Some of the steps in the analysis process are easily parallelizable while others, such as patient clustering, are not. We also intend to investigate more advanced database structures for efficient data management.

## Conclusions

In this study, we develop a visual mining system to support exploratory data analysis of multi-dimensional categorical EMR data. Using CKD as a model disease, a CKD cohort was assembled by automated correlational analysis and human-curated visual evaluation. Our system also shows relevant comorbidities that CKD patients develop over time.

All of this information is combined to produce a Sankey diagram that reveals useful but non-obvious knowledge about the CKD cohort and the expected trajectories of the disease over 13 years. Furthermore, the various parameters governing cohort selection, comorbidity selection, and temporal features are all adjustable by the user and requires no programming knowledge.

Finally, the analysis process is generalizable to any other disease that a user wishes to follow over time and can work with different clustering and filtering algorithms.

## Abbreviations

EMRs: Electronic medical records
CKD: Chronic kidney disease
NHIRD: National Health Insurance Research Database
ICD-9-CM: International Classification of Disease, Ninth Revision, Clinical Modification
CVD: Cerebrovascular disease
CHF: Congestive heart failure
CAD: Coronary artery disease
DM: Diabetes mellitus
GN: Glomerulonephritis
HTN: Hypertension
HD: Hemodialysis
PD: Peritoneal dialysis
RTPL: Renal transplant
PKD: Polycystic kidney disease
SLE: Systemic lupus erythematosus
